# Prediction of tumor recurrence after curative resection in gastric carcinoma based on bcl-2 expression

**DOI:** 10.1186/1477-7819-12-40

**Published:** 2014-02-21

**Authors:** Jianghong Wu, Xiaowen Liu, Hong Cai, Yanong Wang

**Affiliations:** 1 Department of Gastric Cancer and Soft Tissue Sarcoma, Fudan University Shanghai Cancer Center, 270 Dong An Road, Shanghai 200032, China; 2 Department of Oncology, Shanghai Medical College, Fudan University, Shanghai 200032, China

**Keywords:** Gastric cancer, recurrence, clinicopathological factors, bcl-2

## Abstract

**Background:**

There are currently no reliable predictive factors for gastric cancer recurrence. The aim of this study was to evaluate the relationship between bcl-2 expression and risk of gastric cancer recurrence.

**Methods:**

From January 1996 to December 2007, 449 gastric cancer patients who underwent curative resection were retrospectively studied. The expression levels of bcl-2 were examined by immunohistochemistry. Logistic regression was performed to identify independent risk factors for overall recurrence of gastric cancer.

**Results:**

151 patients (33.6%) experienced recurrences. The median time to recurrence was 17.0 months, 113 (74.8%) patients had recurrences within 2 years. Peritoneal recurrence was the most prevalent pattern, followed by hematogenous metastasis in which the liver was the most common site. Depth of invasion, lymph node metastases, and negative expression of bcl-2 were independent risk factors for overall recurrence. The overall survival time of recurrent patients was 22.7 months. The median survival time after recurrence was 6.7 months.

**Conclusion:**

The depth of invasion, lymph node metastases and expression of bcl-2 are independent factors for predicting gastric cancer recurrence.

## Background

Although the incidence of gastric cancer has been declining substantially for several decades, it is still the fourth most common cancer and the second most frequent cause of cancer death [1,2]. Although the majority of patients with early-stage gastric cancer can have long survival times after surgery, approximately 40% of patients die from recurrence, even after undergoing curative surgery [3]. The presence or absence of recurrence is widely considered to be the most important predictor for survival, as the prognosis for patients with recurrence is extremely dismal. Therefore, it is very important to precisely predict the risk of recurrence at an early stage in order to improve prognosis of patients with recurrence. However, it has been demonstrated that routine followup after surgery for gastric cancer is not helpful in detecting recurrence at an early stage [4]. Therefore, it is essential to investigate specific factors that can identify subgroups of patients with more aggressive disease. Recently, some studies have shown that pathological factors such as lymph node status, depth of invasion, and tumor size, play a crucial role in predicting recurrence after gastrectomy [5,6]. However, it is difficult to predict recurrence using common clinicopathological factors. Given that some biological markers, such as oncogenes, tumor-suppressor genes, cell-cycle regulators, and DNA repair genes, are related to tumor genesis, growth, invasion and metastasis, many investigators are searching for new predictive factors among these molecular markers. In a previous study, we evaluated the prognostic value of biological markers such as p53, bcl-2, bax, and c-myc in gastric cancer, and found that bcl-2 was significantly associated with prognosis in gastric cancer patients [7]. Therefore, we hypothesized that bcl-2 expression may reflect tumor characteristics, and thus, serve as a reliable indicator for predicting recurrence. The objective of the current study is to determine whether bcl-2 expression can predict disease recurrence after curative resection in patients with gastric cancer.

## Methods

### Patients

Between January 1996 and December 2007, 4,426 patients with histologically confirmed primary gastric adenocarcinoma underwent gastrectomy at the Department of Gastric Cancer and Soft Tissue Sarcoma in Fudan University Shanghai Cancer Center. Out of these patients, 449 cases were selected. These patients’ specimens were stained immunohistochemically. Data were retrieved from patients’ operative and pathological reports. Follow-up data were obtained by phone, letter, and the outpatient clinical database. Staging was performed according to the Union for International Cancer Control (UICC) *TNM Staging Classification for Carcinoma of the Stomach* (Seventh Edition, 2010) [8]. A followup of all patients was carried out according to our standard protocol (every three months for at least 2 years, every 6 months for the next 3 years, and after 5 years every 12 months for life). The check-up items included physical examination, tumor-marker examination, ultrasound, chest radiography, computed tomographic scan, and endoscopic examination. The range of the followup was from January 1996 to May 2010. The median follow-up time was 76.5 months at the time of analysis. Written informed consent had been obtained from all the patients, and this study was approved by the Ethical Committee of Shanghai Cancer Center of Fudan University. The study was retrospective.

### Immunohistochemical staining

Bcl-2 was detected by the immunohistochemical method. Antibodies were purchased from Dako (Hamburg, Germany). Immunohistochemical staining was performed by the enhanced labeled polymer system method. Paraffin sections were deparaffinised with xylene and hydrated with ethanol/deionized water. Sections were then incubated with methanol/0.3% H_2_O_2_ and blocked with non-specific staining blocking reagent. After overnight incubation at 4°C with anti-bcl-2 antibody (diluted 1:100), sections were treated according to standard immunoperoxidase methods. Negative control sections were subjected to the same procedure, except that the first antibody was replaced by PBS. No positive staining was observed in the controls. The location of staining for bcl-2 was predominantly in cytoplasm and cytomembrane.

### Immunohistochemical staining: scoring

All slices were independently evaluated by two pathologists. The percentage of immunoreactive cells and staining intensities were evaluated. The percentage of immunoreactive cells was graded on a scale of 0 to 4: no staining was scored as 0, 1 to 10% of cells stained scored as 1, 11 to 50% as 2, 51 to 80% as 3, and 81 to 100% as 4. The staining intensities were graded from 0 to 3: 0 was defined as negative, 1 as weak, 2 as moderate, and 3 as strong, respectively. The raw data were converted to an immunohistochemical score (IHS) by multiplying the quantity and intensity scores. An IHS score of 9 to 12 was considered as strong immunoreactivity (+++), 5 to 8 as moderate (++), 1 to 4 as weak (+), and 0 as negative (-). Cases with a score of less than 1 were considered as negative, and those with ≥1 were regarded as positive [9].

### Statistical analysis

Data were presented as the mean ± SD or the median for numeric parameters. The patients’ features and clinical characteristics were analyzed using the two-tailed Student *t*-test or Pearson chi-square test as appropriate. Factors that were deemed of potential importance on univariate analysis were included in the multivariate analysis. Logistic regression was used to estimate related factors for recurrence. The accepted level of significance was *P* < 0.05. Statistical analyses and graphics were performed using the SPSS 13.0 statistical package (SPSS, Inc., Chicago, IL, USA).

## Results

### Clinicopathological characteristics

The study included 307 male and 142 female patients (2.2:1) with a mean age of 57 years. There were 49 (10.9%) early gastric cancers and 400 (89.1%) advanced gastric cancers. According to histological type, well-differentiated tumors were observed in 7 (1.5%) patients, moderately-differentiated in 149 (33.2%) patients, and poorlydifferentiated tumors in the remaining 293 (65.3%) patients. According to Borrmann type, 49 (10.9%) were type 0, 32 (7.1%) type I, 14 (3.1%) type II, 333 (74.2%) type III, and 21 (4.7%) type IV. Of these patients, 147 (32.7%) had tumors located in the upper third of the stomach, 65 (14.5%) had tumors in the middle third, 212 (47.2%) had tumors in the lower third, and 25 (5.6%) had tumors occupying two-thirds or more of the stomach. Lymph node metastasis was observed in 326 patients, and the metastasis rate was 72.6%. The distribution of pathological stage was as follows: 35 (7.8%) patients belonged to stage IA, 34 (7.6%) IB, 50 (11.1%) IIA, 75 (16.7%) IIB, 82 (18.3%) IIIA, 80 (17.8%) IIIB, and 93 (20.7%) IIIC.

### Expression of bcl-2

**Figure 1:**
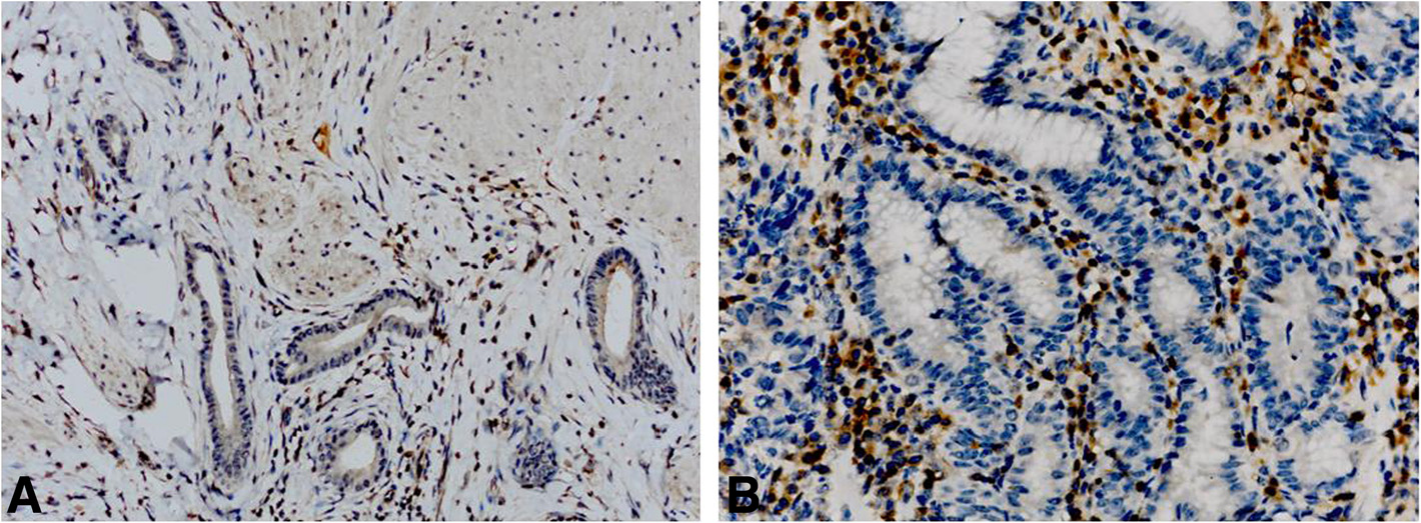
**Positive expression of bcl-2 immunohistochemistry in gastric cancer tissue. A** original magnification ×100, **B** original magnification ×400.

Bcl-2 expression was positive in 21.2% of all gastric cancer tissues. Bcl-2 staining was observed in the cytoplasm and cytomembrane of carcinoma cells (Figure 1). Bcl-2 expression was associated with pathological stage. There was no correlation between bcl-2 and other pathological parameters, such as age, gender, histological type, Borrmann type, tumor location, or tumor size.

### Time to recurrence and recurrence pattern

**Figure 2:**
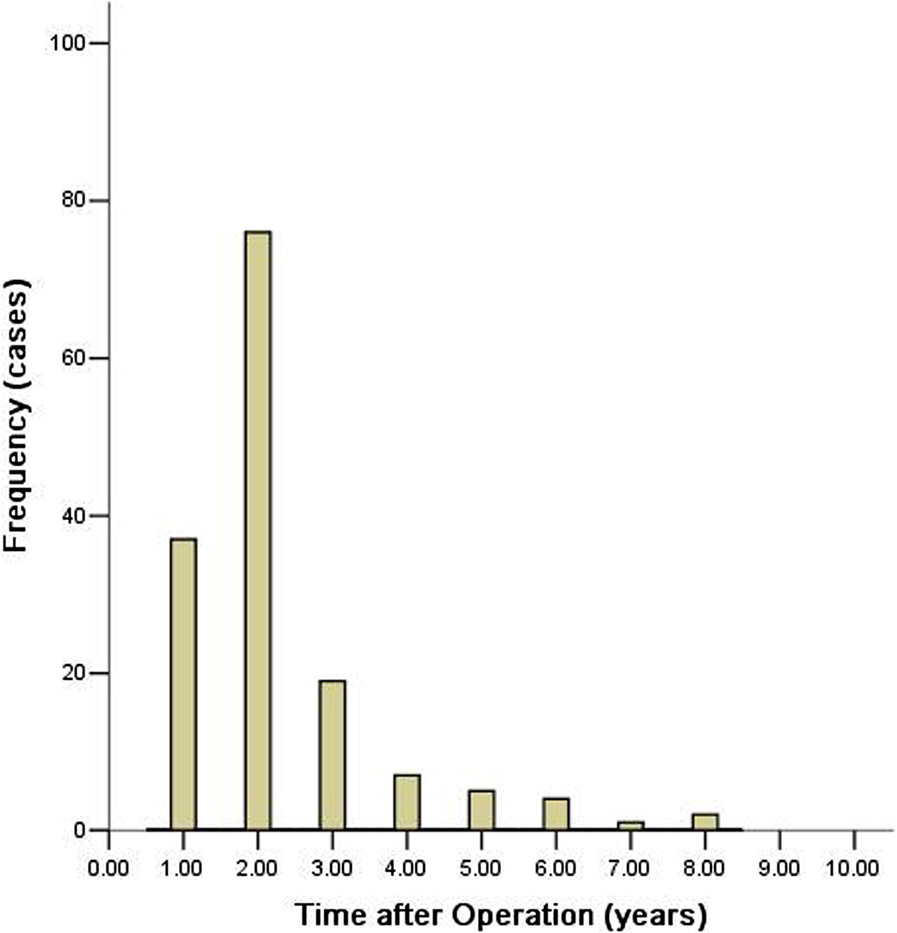
Frequency of recurrence after curative operation by year.

Recurrence occurred in 151 (33.6%) patients during the follow-up period. The median time to recurrence was 17.0 months (range from 2.0 to 91.0 months), and the mean time was 21.1 months. Among patients with recurrence, 113 (74.8%) had recurrence within 2 years. The frequency and rate of recurrence are demonstrated in Figure 2.

**Figure 3:**
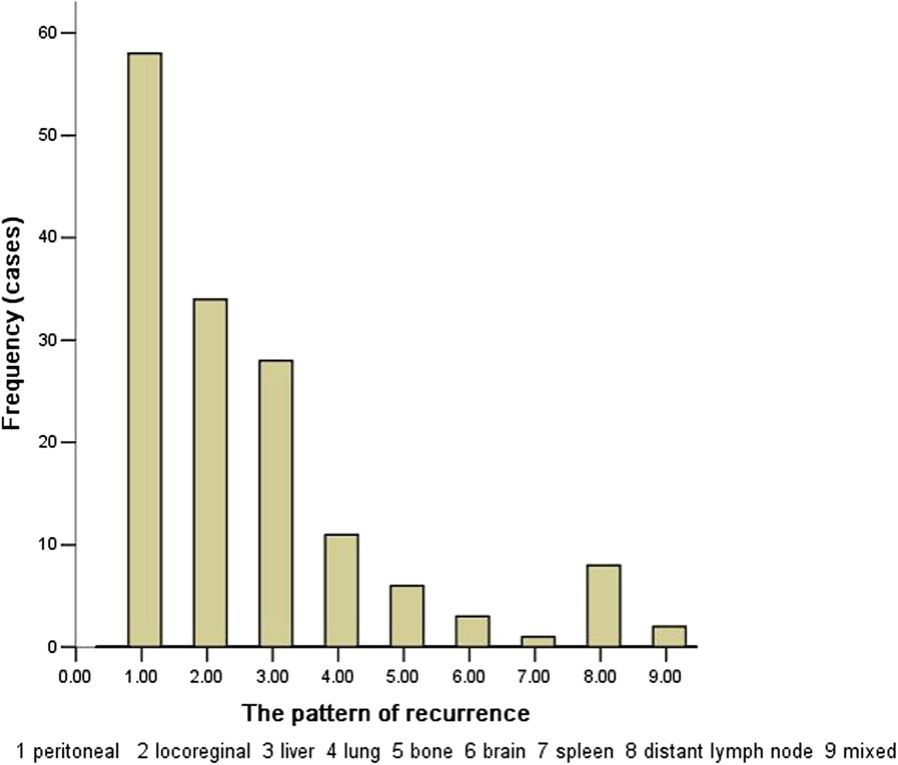
Pattern of recurrence of after curative gastrectomy.

Of the 151 patients with recurrence, 58 (38.4%) were diagnosed with peritoneal recurrence, which was the most prevalent, 49 (32.5%) patients had hematogenous metastases, which was the second most common recurrence pattern, and liver was the most frequently involved organ; it was implicated in 57.1% of all recurrent patients. Other recurrences were loco-regional (n = 34, 22.5%), distant lymph node metastases (n = 8, 5.3%), or at multiple sites (n = 2, 1.3%) (Figure 3).

### Recurrence-related factors

Univariate analysis showed that Borrmann type, depth of invasion, lymph node status, and expression of bcl-2 were associated with overall recurrence, whereas age, gender, histological type, tumor location, and tumor size were not (Table 1). Logistic regression was employed to identify the independent risk factors for overall recurrence. It was demonstrated that depth of invasion, lymph node metastases, and negative expression of bcl-2 were independent predictive factors for overall recurrence (Table 2).

**Table 1:** Univariate analysis of risk factors for recurrence in gastric cancer

	**No recurrence**	**Recurrence**	***P*-value**
	**(n = 298)**	**(n = 151)**	
Age (years), mean ± SD	57.8 ± 11.9	55.8 ± 12.9	0.095
Gender, male/female	203/95	104/47	0.871
Tumor size (cm), mean ± SD	4.8 ± 2.5	5.3 ± 2.7	0.054
Tumor location, upper/middle/lower/two or more	91/38/153/16	56/27/59/9	0.094
Borrmann type, 0/I/II/III/IV	45/22/9/211/11	4/10/5/122/10	0.002
Histology, well/moderate/poorly differentiated	5/101/192	2/48/101	0.899
Depth of invasion, T1 + T2/T3 + T4	94/204	16/135	0.000
Lymph node status, negative/positive	108/190	15/136	0.000
Bcl-2, negative/positive	226/72	128/23	0.029

**Table 2:** Multivariate analysisof risk factors for recurrence in gastric cancer

**Variable**	***P*-value**	**Odds ratio**	**95% CI**
Age	0.116	0.710	0.463, 1.088
Gender	0.775	1.069	0.677, 1.687
Borrmann type	0.334	0.872	0.660, 1.152
Depth of invasion	0.017	0.438	0.222, 0.861
Lymph node status	0.000	3.753	2.040, 6.905
Bcl-2	0.047	0.577	0.336, 0.992

Univariate analysis showed that Borrmann type, depth of invasion, lymph node status, histology, gender, and expression of bcl-2 were associated with peritoneal recurrence. Logistic regression showed that depth of invasion, lymph node status, histology, and bcl-2 were independent predictive factors for peritoneal recurrence. Tumor location and Borrmann type were related to hematogenous metastases, and tumor location was the only independent predictive factor. There was no correlation between clinicopathological parameters and loco-regional recurrence.

### Prognosis of recurrent patients

The overall 5-year survival rate was 52% for all patients. Five-year survival rate was 58.9% and 45.5% in bcl-2 positive and bcl-2 negative patients respectively. In bcl-2 positive patients, pathological stage and tumor location significantly affected prognosis, and pathological stage was an independent prognostic factor. In bcl-2 negative patients, univariate analysis showed that Borrmann type, depth of invasion, lymph node metastases, tumor size, tumor location and pathological stage significantly affected prognosis. Multivariate analysis showed that lymph node metastases, tumor location, and pathological stage were independent prognostic factors.

The median survival time of patients with recurrence was 22.7 months. The median survival time after recurrence was 6.7 months. According to the different recurrence patterns, the median survival time after peritoneal recurrence, hematogenous metastases, loco-regional recurrence, and distant lymph node metastases were 5.7 months (2.6 to 12.4), 7.3 months (2.4 to 11.7), 7.0 months (2.5 to 17.1), and 6.1 months (3.9 to 7.9), respectively. Patients with peritoneal and distant lymph node recurrence had a shorter survival time; however this was not statistically significant.

## Discussion

The main findings of this study were that (1) peritoneal recurrence was the most prevalent recurrence pattern after curative surgery, and (2) the depth of invasion, lymph node metastases and negative expression of bcl-2 were independent risk factors for overall recurrence.

Gastric cancer is one of the most common malignancies worldwide. Although the prognosis of patients with gastric cancer has improved as a result of better diagnostic techniques and therapeutic strategies, gastric cancer is still the second leading cause of cancer-related deaths [2]. The dismal prognosis of gastric cancer is due principally to the frequency of recurrence and metastasis. Therefore, it is very important to clarify the pattern of recurrence and the risk factors for recurrence, which can contribute to the conduct of postoperative clinical practice and followup.

In our study, 151 (33.6%) patients had recurrence during the follow-up period. The mean time to recurrence was 21.1 months; 113 patients (74.8%) had recurrence within 2 years. This result shows that most gastric cancer recurrence occurs within 2 years, and is consistent with other studies [10-12]. Yoo *et al.*[12] reported 508 patients with recurrence after curative resection for gastric cancer and showed that the mean length of time to recurrence was 21.8 months. Therefore, it was necessary to reinforce the followup within 2 years after curative resection.

It has been reported that the most common recurrence patterns are hematogenous recurrence and peritoneal dissemination in gastric cancer [3,12-14]. In our series, peritoneal recurrence was the most common pattern, accounting for 38.4% of all patients with recurrence, followed by hematogenous metastases (32.5%). Our results are consistent with previous studies [3,12]. Maehara *et al.* reported [15] that the peritoneal pattern accounted for 50% of cases, hematogenous recurrence for 40% of cases, and loco-regional recurrence for 20%. However, there are still some disputes. An Italian study showed that the most common recurrence pattern was loco-regional, accounting for 45% of all recurrent cases [16]. This disparity indicated that there are some differences in recurrence patterns between Eastern and Western countries. It is possible that the low incidence of local recurrence in Eastern studies is due to the extensive use of D2 lymph-node dissection.

Recurrence is frequent in gastric cancer patients after undergoing radical gastrectomy. Therefore, it is important to identify patients at a high risk of recurrence. In this study, univariate analysis showed that Borrmann type, depth of invasion, and lymph node status were associated with overall recurrence. Logistic regression demonstrated that depth of invasion and lymph node metastases were independent risk factors for overall recurrence. Consistent with our findings, previous studies have showed that traditional clinicopathological factors such as serosal exposure, histological type, depth of invasion, and lymph node metastasis were risk factors for recurrence [17-19]. However, traditional clinicopathological factors are sometimes inadequate for prediction of recurrence. A number of studies have indicated that biomarkers, such as tumor markers [20,21], vascular endothelial growth factor (VEGF) family [22,23], E-cadherin [24,25], and microRNAs [26], might be useful for predicting recurrence of gastric cancer. Bcl-2 located at chromosome 18q21, and encodes a 26-kDa protein localized mainly in the mitochondrial membrane [27]. The bcl-2 gene was first described as an oncogene in follicular lymphoma. Some studies have shown that overexpression of bcl-2 suppressed cellular proliferation and was associated with less aggressive biological behaviour [28,29]. However, the value of bcl-2 in predicting gastric cancer recurrences is still unclear. In a previous study, we found that bcl-2 was significantly associated with prognosis in gastric cancer patients [7]. Therefore, we hypothesized that bcl-2 might reflect the biological nature of the tumor, and would serve as a reliable indicator for predicting recurrence. According to our results, the negative expression of bcl-2 is an independent risk factor for gastric cancer recurrence. The exact mechanism is still unclear. It is possible that bcl-2 affects gastric cancer recurrence by regulating the tumor cell biological behaviour. Saegusa *et al.*[28] reported that the majority of bcl-2+ cancer cells are in a nonproliferative state, and the average expression of Ki-67 labeling index and apoptotic labeling index in bcl-2+ foci are significantly lower than that in bcl-2 foci. Aizawa *et al.*[30] showed that the expression of bcl-2 in advanced gastric cancer is associated with a lower apoptotic index and a better prognosis. Pietenpol *et al.*[31] reported that over-expression of bcl-2 inhibits the growth of several solid-tumor cell lines.

## Conclusion

In conclusion, the present study demonstrated that peritoneal recurrence was the most common recurrence pattern after curative gastrectomy. Depth of invasion, lymph node metastases, and negative expression of bcl-2 predict greater risk of recurrence. These findings may provide some useful information for followup in gastric cancer patients who have undergone surgery for resection. Specifically, it is necessary to conduct an intensive follow-up plan for gastric cancer patients with negative expression of bcl-2. However, the small scale and the retrospective design were shortcomings of the current study. Further large-scale, prospective studies should be performed to validate the usefulness of bcl-2 in detecting tumor recurrence in patients with gastric cancer.

## Competing interests

The authors declare that they have no competing interests.

## Authors’ contributions

JHW, XWL and YNW conceived and designed the experiments. JHW and XWL performed the experiments. JHW, XWL and HC analyzed the data. HC and YNW contributed reagents/materials/analysis tools. JHW and XWL wrote the paper. All authors read and approved the final manuscript.

## References

[B1] Shibata A Parsonnet J Schottenfeld D, Fraumeni JF Stomach cancer Cancer epidemiology and prevention 2006 3 New York: Oxford University Press 707 720

[B2] Parkin DM Bray F Ferlay J Pisani P Global cancer statistics, 2002 CA Cancer J Clin 2005 55 74 108 10.3322/canjclin.55.2.74 15761078

[B3] Moriguchi S Maehara Y Korenaga D Sugimachi K Nose Y Risk factors which predict pattern of recurrence after curative surgery for patients with advanced gastric cancer SurgOncol 1992 1 341 346 10.1016/0960-7404(92)90034-i 1341269

[B4] Bohner H Zimmer T Hopfenmuller W Berger G Buhr HJ Detection and prognosis of recurrent gastric cancer-is routine follow-up after gastrectomy worthwhile? Hepatogastroenterology 2000 47 1489 1494 11100384

[B5] Kim JH Kim HS Seo WY Nam CM Kim KY Jeung HC Lai JF Chung HC Noh SH Rha SY External validation of nomogram for the prediction of recurrence after curative resection in early gastric cancer Ann Oncol 2012 23 361 367 10.1093/annonc/mdr118 21566150

[B6] Chou HH Kuo CJ Hsu JT Chen TH Lin CJ Tseng JH Hwang TL Jan YY Clinicopathologic study of node-negative advanced gastric cancer and analysis of factors predicting its recurrence and prognosis Am J Surg 2013 205 623 630 10.1016/j.amjsurg.2012.04.014 23036602

[B7] Liu X Cai H Huang H Long Z Shi Y Wang Y The prognostic significance of apoptosis-related biological markers in Chinese gastric cancer patients PLoS One 2011 6 e29670 10.1371/journal.pone.0029670 22216342 PMC3247293

[B8] Sobin LH Gospodarowicz MK Wittekind C International Union against Cancer (UICC) TNM Classification of Malignant Tumor 2010 7 New York: Wiley-Liss

[B9] Soslow R Dannenberg A Rush D Woerner BM Khan KN Masferrer J Koki AT COX-2 is expressed in human pulmonary, colonic, and mammary tumors Cancer 2000 89 2637 2645 10.1002/1097-0142(20001215)89:12<2637::AID-CNCR17>3.0.CO;2-B 11135226

[B10] Lai JF Kim S Kim K Li C Oh SJ Hyung WJ Rha SY Chung HC Choi SH Wang LB Noh SH Prediction of recurrence of early gastric cancer after curative resection Ann SurgOncol 2009 16 1896 1902 10.1245/s10434-009-0473-x 19434457

[B11] Shiraishi N Inomata M Osawa N Yasuda K Adachi Y Kitano S Early and late recurrence after gastrectomy for gastric carcinoma. Univariate and multivariate analyses Cancer 2000 89 255 261 10.1002/1097-0142(20000715)89:2<255::AID-CNCR8>3.0.CO;2-N 10918153

[B12] Yoo CH Noh SH Shin DW Choi SH Min JS Recurrence following curative rection for gastric carcinoma Br J Surg 2000 87 236 242 10.1046/j.1365-2168.2000.01360.x 10671934

[B13] Folli S Dente M Dell’Amore D Gaudio M Nanni O Saragoni L Vio A Early gastric cancer: prognostic factors in 223 patients Br J Surg 1995 82 952 956 10.1002/bjs.1800820732 7648118

[B14] Lee HJ Kim YH Kim WH Lee KU Choe KJ Kim JP Yang HK Clinicopathological analysis for recurrence of early gastric cancer Jpn J ClinOncol 2003 33 209 214 10.1093/jjco/hyg042 12865463

[B15] Maehara Y Hasuda S Koga T Tokunaga E Kakeji Y Sugimachi K Postoperative outcome and sites of recurrence in patients following curative resection of gastric cancer Br J Surg 2000 87 353 357 10.1046/j.1365-2168.2000.01358.x 10718807

[B16] Roviello F Marrelli D de Manzoni G Morgagni P Di Leo A Saragoni L De Stefano A Italian Research Group for Gastric Cancer Prospective study of peritoneal recurrence after curative surgery for gastric cancer Br J Surg 2003 90 1113 1119 10.1002/bjs.4164 12945079

[B17] Li F Zhang R Liang H Liu H Quan J The pattern and risk factors of recurrence of proximal gastric cancer after curative resection J Surg Oncol 2013 107 130 135 10.1002/jso.23252 22949400

[B18] Ichiyoshi Y Toda T Minamisono Y Nagasaki S Yakeishi Y Sugimachi K Recurrence in early gastric cancer Surgery 1990 107 489 495 2333591

[B19] Sano T Sasako M Kinoshita T Maruyama K Recurrence of early gastric cancer. Follow-up of 1475 patients and review of the Japanese literature Cancer 1993 72 3174 3178 10.1002/1097-0142(19931201)72:11<3174::AID-CNCR2820721107>3.0.CO;2-H 8242540

[B20] Emoto S Ishigami H Yamashita H Yamaguchi H Kaisaki S Kitayama J Clinical significance of CA125 and CA72-4 in gastric cancer with peritoneal dissemination Gastric Cancer 2012 15 154 161 10.1007/s10120-011-0091-8 21892754

[B21] Ito S Nakanishi H Kodera Y Mochizuki Y Tatematsu M Yamamura Y Prospective validation of quantitative CEA mRNA detection in peritoneal washes in gastric carcinoma patients Br J Cancer 2005 93 986 992 10.1038/sj.bjc.6602802 16205696 PMC2361668

[B22] Wang X Cao W Mo M Wang W Wu H Wang J VEGF and cortactin expression are independent predictors of tumor recurrence following curative resection of gastric cancer J SurgOncol 2010 102 325 330 10.1002/jso.21644 20589712

[B23] Aoyagi K Kouhuji K Yano S Miyagi M Imaizumi T Takeda J Shirouzu K VEGF significance in peritoneal recurrence from gastric cancer Gastric Cancer 2005 8 155 163 10.1007/s10120-005-0329-4 16086118

[B24] Zhong XY Zhang LH Jia SQ Shi T Niu ZJ Du H Zhang GG Hu Y Lu AP Li JY Ji JF Positive association of up-regulated Cripto-1 and down-regulated E-cadherin with tumour progression and poor prognosis in gastric cancer Histopathology 2008 52 560 568 10.1111/j.1365-2559.2008.02971.x 18312357

[B25] Chen HC Chu RY Hsu PN Hsu PI Lu JY Lai KH Tseng HH Chou NH Huang MS Tseng CJ Hsiao M Loss of E-cadherin expression correlates with poor differentiation and invasion into adjacent organs in gastric adenocarcinomas Cancer Lett 2003 201 97 106 10.1016/j.canlet.2003.07.007 14580691

[B26] Zhang X Yan Z Zhang J Gong L Li W Cui J Liu Y Gao Z Li J Shen L Lu Y Combination of hsa-miR-375 and hsa-miR-142-5p as a predictor for recurrence risk in gastric cancer patients following surgical resection Ann Oncol 2011 22 2257 2266 10.1093/annonc/mdq758 21343377

[B27] Skinnider BF Horsman DE Dupuis B Gascoyne RD Bcl-6 and Bcl-2 protein expression in diffuse large B-cell lymphoma and follicular lymphoma: correlation with 3q27 and 18q21 chromosomal abnormalities Hum Pathol 1999 30 803 808 10.1016/S0046-8177(99)90141-7 10414499

[B28] Saegusa M Takano Y Okayasu I Bcl-2 expression and its association with cell kinetics in human gastric carcinomas and intestinal metaplasia J Cancer Res ClinOncol 1995 121 357 363 10.1007/BF01225688 PMC12201682 7797601

[B29] Nakanishi H Ohsawa M Naka N Uchida A Ochi T Aozasa K Immunohistochemical detection of bcl-2 and p53 proteins and apoptosis in soft tissue sarcoma: their correlations with prognosis Oncology 1997 54 238 244 10.1159/000227695 9143406

[B30] Aizawa K Ueki K Suzuki S Yabusaki H Kanda T Nishimaki T Suzuki T Hatakeyama K Apoptosis and Bcl-2 expression in gastric carcinoma: correlation with clinicopathological variables, p53 expression, cell proliferation and prognosis Int J Oncol 1999 14 85 91 9863013 10.3892/ijo.14.1.85

[B31] Pietenpol JA Papadopoulos N Markowitz S Willson JK Kinzler KW Vogelstein B Paradoxical inhibition of solid tumor cell growth by bcl-2 Cancer Res 1994 54 3714 3717 8033089

